# Solubility measurement of verapamil for the preparation of developed nanomedicines using supercritical fluid

**DOI:** 10.1038/s41598-023-44280-7

**Published:** 2023-10-10

**Authors:** Nadia Esfandiari, Nedasadat Saadati Ardestani, Ratna Surya Alwi, Adrián Rojas, Chandrasekhar Garlapati, Seyed Ali Sajadian

**Affiliations:** 1 Department of Chemical Engineering, Marvdasht Branch, Islamic Azad University, Marvdasht, Iran; 2 Nanotechnology Research Center, Research Institute of Petroleum Industry (RIPI), P.O. Box 14857-336, Tehran, Iran; 3 National Research and Innovation Agency (BRIN), Jl. Raya Jakarta-Bogor KM 46, Cibinong, Indonesia; 4 Center for the Development of Nanoscience and Nanotechnology (CEDENNA), 9170124 Santiago, Chile; 5 Packaging Innovation Center (LABEN), Department of Science and Food Technology, Faculty of Technology, University of Santiago of Chile (USACH), Obispo Umaña 050, 9170201 Santiago, Chile; 6 Department of Chemical Engineering, Pondicherry Technological University, Puducherry, 605014 India; 7 Department of Chemical Engineering, Faculty of Engineering, University of Kashan, Kashan, 87317-53153 Iran; 8 South Zagros Oil and Gas Production, National Iranian Oil Company, Postal Code, Shiraz, 7135717991 Iran

**Keywords:** Chemical engineering, Chemistry

## Abstract

A static method is employed to determine the solubilities of verapamil in supercritical carbon dioxide (SC-CO_2_) at temperatures between 308 and 338 K and pressures between 12 and 30 MPa. The solubility of verapamil in SC-CO_2_ expressed as mole fraction are in the range of 3.6 × 10^–6^ to 7.14 × 10^–5^. Using four semi-empirical density-based models, the solubility data are correlated: Chrastil, Bartle, Kumar–Johnston (K–J), and Mendez-Santiago and Teja (MST), two equations of state (SRK and PC-SAFT EoS), expanded liquid models (modified Wilson's models), and regular solution model. The obtained results indicated that the regular solution and PC-SAFT models showed the most noteworthy exactness with *AARD%* of 1.68 and 7.45, respectively. The total heat, vaporization heat, and solvation heat of verapamil are calculated at 39.62, 60.03, and − 20.41 kJ/mol, respectively. Regarding the poor solubility of verapamil in SC-CO_2_, supercritical anti-solvent methods can be an appropriate choice to produce fine particles of this drug.

## Introduction

Verapamil is one of the common calcium channel blockers (CCB) applied to treat excessive blood pressure, various forms of irregular heartbeats (arrhythmia), and angina (chest pain associated with the heart). It reduces blood pressure by relaxing blood vessels, which lessens the stress on the heart^1^. Verapamil is a Class I drug which has good gastrointestinal membrane absorption (> 90%) after oral treatment with poor bioavailability (20–35%)^2^ due to is practically insoluble in water^3^. The drug's particle size can be decreased to reduce the specific effective surface area that will be exposed to the solvent to maximize its solubility and bioavailability absorption. In recent years, supercritical fluids (SCFs), especially SC-CO_2_, have been used to reduce the size of drugs particles^4^. The choice of an adequate SCFs-based method to reduce a drug particle depends on how well the drug substance dissolves in SC-CO_2_. Several medication micronization techniques employing SC-CO_2_ are available, depending on the SCF's function (solute, solvent, or anti-solvent)^5^. The rapid expansion of the supercritical solutions (RESS) technique is suggested if the medicine has strong solubility in SC-CO_2_^6^. If the drug does not have good solubility, SC-CO_2_ is utilized as an anti-solvent. Supercritical antisolvent (SAS)^7^, gas antisolvent (GAS)^8–11^, solution enhanced dispersion by supercritical (SEDS)^12,13^, and aerosol solvent extraction system (ASES)^14,15^ are the techniques used in this scenario. When the supercritical fluid is used as a solute, the gas saturated solution (PGSS) is utilized^16^.

The solubility of numerous pharmacological compounds has been studied via semi-empirical models. Investigations into the solubility of glibenclamide in SC-CO_2_ are done between 308 and 338 K and 12 and 30 MPa. The glibenclamide solubility is modeled using semi-empirical models from Chrastil, Bartle et al., Mendez-Santiago and Teja (MST), Sparks et al., Bian et al., and Sodeifian et al., as well as PR-EoS with vdW2 mixing rule^17^. Experimental measurements are made to determine the mole fraction solubility of favipiravir, which ranged from 3.0 × 10^–6^ to 9.05 × 10^–4^. The solubility of favipiravir is investigated by density-based models (Chrastil, Garlapati and Madras, Sparks et al., Sodeifian et al., K–J and Keshmiri et al.), SRK EoS with quadratic mixing rules, and (c) extended liquid theory (modified Wilson model)^18^. Lacosamide's experimental solubility results showed that its mole fractions in SC-CO_2_ are assessed as having a minimum and maximum solubility at 338 K and pressures of 12 and 30 MPa, respectively. The modeling outcomes showed that the models' accuracy in describing the solubility of lacosamide declined in the following order: the modified Wilson model, PC-SAFT EoS, PR EoS, semi-empirical model K–J, MST, Bartle et al., and Chrastil ^19^. MST and PC-SAFT are the most accurate models for fludrocortisone acetate solubility in SC-CO_2_^20^. Experimental work is done to determine the solubility of ketoconazole in SC-CO_2_ at the temperature and pressure limits of 308 K and 12 MPa, respectively. Meanwhile, the solubility of ketoconazole is correlated using the semi-empirical models^21^.

This research examined the solubility of verapamil in SC-CO_2_ at pressures between 12 and 30 MPa and temperatures between 308 and 338 K. For this purpose, solubility data are correlated using equations of state (Soave Redlich Kwong and PC-SAFT), expanded liquid models (modified Wilson's models), and the regular solution model in addition to semi-empirical models Chrastil, K–J, Bartle, and MST. Calculations are used to determine the mean absolute deviation (*AARD%*) and the corrected correlation coefficient (*R*_*adj*_). The models' validity is tested using these two parameters.

## Experimental

### Materials

Pure verapamil is supplied by the Sobhan Darou. Moreover, 99.99% pure methanol is purchased from Merck. Orders for CO_2_ with a 99.99% purity level are placed to Oxygen Novin Company. Table 1 shows the properties of verapamil.

**Table 1:** The properties of substances.

Compound/Formula	Supplier	CAS Number	Structure	Mass Purity	Analysis method	M_w_ (g/mol)^c^	T_m_ (K)^d^	λ (nm)^e^
Verapamil C_27_H_38_N_2_O_4_	Sobhan Darou	52–53-9		0.999 <	HPLC^a^	454.6	414.05	275
Methanol	Merck Co	67–56-1		0.999	GC^b^			
CO_2_	Oxygen Novin Co	124–38-9		0.999	GC^b^			

^a^High-performance liquid chromatography.

^b^Gas chromatography.

^c^Molecular weight.

^d^Melting temperature.

^e^Maximum wavelength.

^c,d^http://www.chemspider.com.

### Experimental apparatus

**Figure 1:**
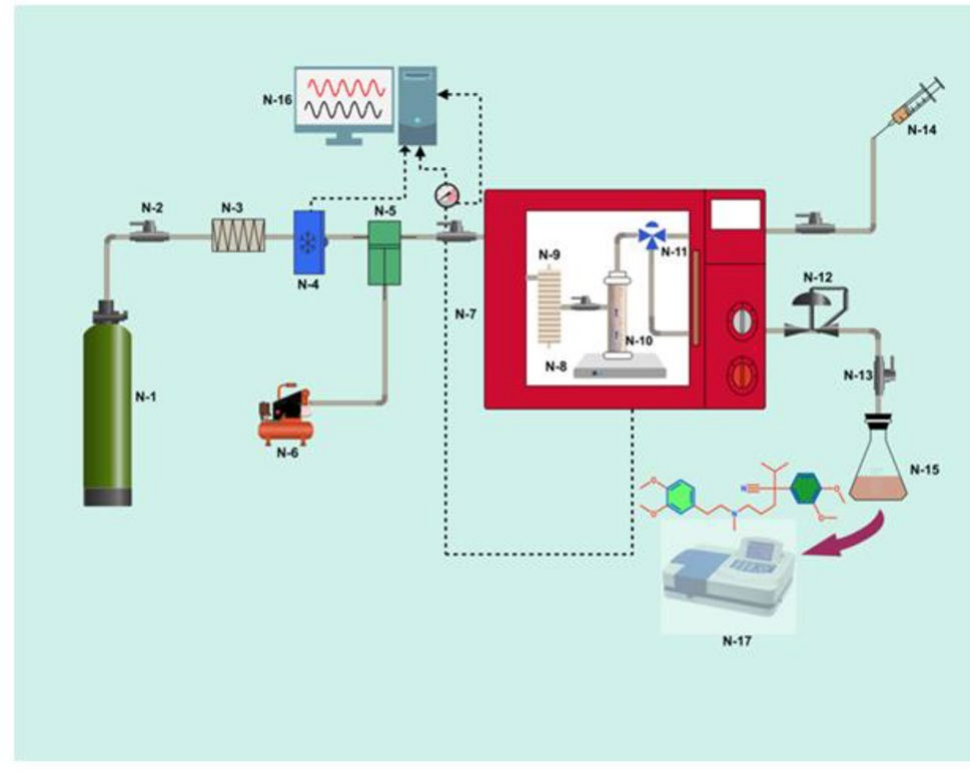
Test devices of verapamil solubility measurement. Equipment includes Chamber of CO_2_ (N-1), Needle valve (N-2), Filter (N-3), Refrigerator (N-4), Pump (N-5), Air compressor (N-6), Oven (N-7), Stirrer (N-8), Coil (N-9), Cell (N-10), 3-position valve (N-11), Back pressure (N-12), Micrometer valve (N-13), Syringe (N-14), Gathering vial (N-15), Panel (N-16), and UV (N-17).

The solubility measurement setup of verapamil is shown in Fig. 1. The equipment list includes a chamber of CO_2_ (N-1), Needle valve (N-2), Filter (N-3), Refrigerator (N-4), Pump (N-5), Air compressor (N-6), Oven (N-7), Stirrer (N-8), Coil (N-9), Cell (N-10), 3-position valve (N-11), Back pressure (N-12), Micrometer valve (N-13), Syringe (N-14), Gathering vial (N-15), and Panel (N-16). A molecular sieve as filter is used to separate the impurities of CO_2_. A refrigeration unit at – 10 °C is used to liquefy CO_2_. A high-pressure pump is used to modify the pressure of the liquid CO_2_ and an oven is used to change the temperature of the liquid CO_2_. 2000 mg of verapamil is loaded in the equilibrium cell (300 mL). Subsequently, the pressure in the equilibrium cell is increased with the addition of CO_2_. To achieve the equilibrium condition, the cell is preserved at the desired temperature and pressure for 140 min. The 3-position valve is used to upload a saturated SC-CO_2_ sample into the sample assemble (500 µL ± 0.6% volume). Then, the saturated SC-CO_2_ sample is depressurized into a specific methanol volume. A valve is used to control the pressure drop to prevent the solvent dispersion. Finally, methanol is used to clean the line. A UV-V spectrophotometer is used to measure the verapamil concentration in the methanol solutions utilizing a calibration curve made using a 25 µg/mL primary solution. When the main solution is diluted, solutions with various concentrations are constructed. The following equations are used to calculate the number of verapamil (*n*_*solute*_) and CO_2_ moles (*n*_*CO2*_) in the taste loop:

1 $${n}_{solute}=\frac{{C}_{s}\left(\frac{g}{L}\right)\times {V}_{s}\left(L\right)}{{M}_{s}\left(\frac{g}{mol}\right)}$$

2 $${n}_{c{o}_{2}}=\frac{{V}_{l}\left(L\right)\times \rho \left(\frac{g}{L}\right)}{{M}_{C{O}_{2}}\left(\frac{g}{mol}\right)}$$

where ${V}_{l}$(L) and ${V}_{s}(L)$ are the volumes of the sampling loop and the gather vial, respectively, and ${C}_{s}$ is the verapamil concentration (g/L) in the collection vial.

In various test conditions, the mole fraction of verapamil, *y*_2_, is computed as follows:

3 $${y}_{2}=\frac{{n}_{solute}}{{n}_{solute}+{n}_{{co}_{2}}}$$

The verapamil solubility in SC-CO_2_ is calculated by Eqs. (4) or (5).

4 $$S \left(\frac{g}{L}\right)=\frac{{C}_{s}\left(\frac{g}{L}\right)\times {V}_{s}\left(L\right)}{{V}_{l}\left(L\right)}$$

5 $$S=\frac{\rho \times {M}_{solute}{\times y}_{2}}{{M}_{C{O}_{2}}\times \left(1-{y}_{2}\right)}$$

## Modeling

To modeling the solubility of verapamil in scCO_2_ are employed the semi-empirical density-based models (Chrastil, K–J, MST, and Bartle et al.), (ii) EoS-based SRK and PC-SAFT, (iii) expanded liquid theory (modified Wilson's model), and (iv) regular solution models. The adjustable parameters of the models are optimized through the simulated annealing (SA) algorithm in MATLAB software.

### Semi-empirical density-based models

Table 2 displays the semi-empirical density-based models. These models have three variables that can be estimated using empirical data, y is the verapamil mole fraction and ρ is the density of SC-CO_2_ in semi-empirical models. *P*_*ref*_ and *ρ*_*ref*_ in the Bartle et al. model are equivalent to 700 kg/m^3^ and 0.1 MPa, respectively.

**Table 2:** The formula of semi-empirical model^a^.

Name	Formula
Chrastil ^47^	$ln S={a}_{0}lnln \rho +{a}_{1}+\frac{{a}_{2}}{T}$
Bartle et al*.* ^48^	$ln\left(\frac{{y}_{2}P}{{P}_{ref}}\right)={a}_{0}+{a}_{1}\left(\rho -{\rho }_{ref}\right)+\frac{{a}_{2}}{T}$
MST ^49^	$Tln \left({y}_{2}P\right)={a}_{0}+{a}_{1}\rho +{a}_{2}T$
K–J^50^	$ln{y}_{2}={a}_{0}+{a}_{1}\rho +\frac{{a}_{2}}{T}$

^a^a_0_ − a_2_, adjustable parameters of models.

The $AARD\%$ (Eq. (6)) and *R*_*adj*_ (Eq. (7)) are utilized to optimize the adjustable parameters of semi-empirical models^22,23^:

6 $$AARD\%=\frac{100}{{N}_{t}-{N}_{f}}{\sum }_{i=1}^{{N}_{t}}\frac{\left|{y}_{2}^{cal}-{y}_{2}^{exp}\right|}{{y}_{2}^{exp}}$$

7 $$R_{adj} = \sqrt {\left| {R^{2} - ({{Q(1 - R^{2} )} \mathord{\left/ {\vphantom {{Q(1 - R^{2} )} {(N - Q - 1))}}} \right. \kern-0pt} {(N - Q - 1))}}} \right|}$$

Here ${N}_{t}$ and ${N}_{f}$ are the number of report points in each series and the number of fitted parameters for each model. The report data in each series and the number of self-reliant variables in any equation are denoted by the letters *N* and *Q*, respectively.

### Equation of state-based (EoS) models

When two phases are in equilibrium, the temperature, pressure, and fugacity of the two phases must be equal to each other. Equation (8) can be used to demonstrate that verapamil and SC-CO_2_ have equal fugacity as follows:

8 $${f}_{2}^{SC-C{O}_{2}}={f}_{2}^{solid}$$

In this model, it is assumed that the solid phase is pure, CO_2_ is insoluble in the solid phase, and pressure does not affect the solute's molar volume. verapamil's solubility in SC-CO_2_ is found using the following formula:

9 $${y}_{2}=\frac{{P}_{2}^{sub}(T)}{P}\frac{{\varnothing }_{2}^{sat}(T)}{{\varnothing }_{2}(T,P,y)}exp\left[\frac{{v}_{2}^{s}(P-{P}_{2}^{sub}\left(T\right))}{RT}\right]$$

where ${P}_{2}^{sub}$ is the sublimation pressure of solute (verapamil), as determined by the Ambrose–Walton method^24^, ${\varnothing }_{2}$ is the verapamil fugacity coefficient in the supercritical carbon dioxide, ${v}_{2}^{s}$ is the molar volume of the verapamil, as calculated by Immirzi–Perini method^25^, ${\varnothing }_{2}$ is the verapamil fugacity coefficient in SC-CO_2_ is defined by EoS as Eq. (10):

10 $$RTln {\varnothing }_{i}=-RTlnZ+\left[{\left(\frac{\partial P}{\partial {n}_{i}}\right)}_{T,V,{n}_{j}\ne {n}_{i}}-\frac{RT}{V}\right]dV$$

Here, the fugacity coefficient is calculated using the SRK and PC-SAFT EoS. The Marrero and Gani method is used to compute the critical properties, and normal boiling point^26^, the acentric factor ($\omega$) is calculated using the equivalent state approach of Ambros-Walton^27^, solid molar volume (v_s_)^25^, and sublimation pressure^27^ of solids in different temperature are shown in Table 3.

**Table 3:** The physicochemical characteristic of verapamil.

Material	T_b_ (K)	T_c_ (K)	P_c_ (bar)	ω	V_s_ (cm^3^/mol)	T(K)			
						308	318	328	338
						P_sub_^d^ (MPa)			
Verapamil	738.84^a^	1009^a^	8.833^a^	0.1089^b^	395.12^c^	4.22 × 10^–7^	8.69 × 10^–7^	1.708 × 10^–6^	3.211 × 10^–6^

(*T*_*b*_): Boiling Point; (*T*_*c*_): critical point; (*P*_*c*_): critical pressure; (*ω*): acentric factor; (*V*_*s*_): solid molar volume; (*P*_*sub*_): Sublimation Pressure; ^a^Marrero and Gani procedure^26^. ^b,d^Ambrose–Walton procedure^27^. ^c^Immirzi–Perini procedure^25^.

#### Soave Redlich Kwong equation of state

The relation of SRK-EoS and parameters (*a*, *b*, $\alpha$) are shown in Eqs. (11–15)^28^.

11 $$P=\frac{RT}{v-b}-\frac{a\left(T\right)}{v\left(v+b\right)}$$

12 $$a\left(T\right) =\frac{0.42747{ R}^{2}{ T}_{c}^{2}}{{P}_{c}}\times \alpha \left({T}_{r,\omega }\right)$$

13 $$b =\frac{0.08664R{ T}_{c}}{{P}_{c}}$$

14 $$\alpha \left({T}_{r,\omega }\right) ={ \left[1+ m \left(1 -{ T}_{r}^{0.5}\right)\right] }^{2}$$

15 $$m = 0.480 +1.574 \omega - 0.176{ \omega }^{2}$$

The mixing rule of parameters is defined as:

16 $$a{}_{m} = \sum\limits_{i = 1}^{2} {\sum\limits_{j = 1}^{2} {x_{i} x_{j} a_{ij} } }$$

17 $$b{}_{m} = \sum\limits_{i = 1}^{2} {\sum\limits_{j = 1}^{2} {x_{i} x_{j} b_{ij} } }$$

Here, ${a}_{ij}$ and ${b}_{ij}$ are calculated as follows^28^:

18 $${a}_{ij}={\left({a}_{i}{a}_{j}\right)}^{0.5}\left(1-{k}_{ij}\right)$$

19 $${b}_{ij}=\left(\frac{{b}_{i}+{b}_{j}}{2}\right)\left(1-{l}_{ij}\right)$$

#### PC-SAFT equation of state

The PC-SAFT can be explained by Eq. (20) ^29^:

20 $${\check{a}} = \frac{A}{NKT} = a^{id} + a^{hc} + a^{disp}$$

Here, ${a}^{id}, {a}^{hc}, \mathrm{and} {a}^{disp}$ are the contribution of ideal gas contribution, hard-sphere chain, and dispersion forces. In this study, the contribution of molecular association resulting from hydrogen bonding in the system is not considered in Eq. (20). This is because the solubility of Verapamil in SC-CO_2_ is minimal (less than 0.001 in mole fraction), as described the result section. Accordingly, the effect of the self-association between API molecules is considered as small. Therefore, only the following three pure-component parameters of PC-SAFT are used in this study. In several articles, authors applied this equation for calculating APIs solubility in SC-CO_2_^30–32^. The value of Helmholtz energy for the N-component of non-associating chains is obtained in Eq. (21):

21 $${\widetilde{a}}^{res}={\widetilde{a}}^{hc}+{\widetilde{a}}^{disp}$$

The hard-chain reference contribution is written as:

22 $${\widetilde{a}}^{hc}=\underline{m}{\widetilde{a}}^{hs}-{\sum }_{i=1}^{N}{y}_{i}\left({m}_{i}-1\right)ln{g}_{ii}^{hs}\left({\sigma }_{ii}\right)$$

$\underline{m}$ is the mean segment number in the combination is calculated by Eq. (23):

23 $$\underline{m}={\sum }_{i=1}^{N}{y}_{i}{m}_{i}$$

##### Dispersion contribution

The dispersion force contribution is defined by Eq. (24) ^33^.

24 $$\begin{gathered} \tilde{a}^{disp} = - 2\pi \rho \left[ {l_{1,xk} \underline{{m^{2} \varepsilon \sigma^{3} }} + l_{1} \underline{{\left( {m^{2} \varepsilon \sigma^{3} } \right)_{xk} }} } \right] - \pi \rho \left\{ {\left[ {m_{k} C_{1} l_{2} + \underline {m} C_{1,xk} l_{2} + \underline {m} C_{1} l_{2,xk} } \right] \times } \right. \hfill \\ \left. {\underline{{m^{2} \varepsilon^{2} \sigma^{3} }} + \underline {m} C_{1} l_{2} \underline{{\left( {m^{2} \varepsilon^{2} \sigma^{3} } \right)_{xk} }} } \right\} \hfill \\ \end{gathered}$$

The mixing rule for segment diameter $\left({\sigma }_{ij}\right)$ and energy $\left({\varepsilon }_{ij}\right)$ are defined as:

25 $${\sigma }_{ij}=\frac{1}{2}\left({\sigma }_{i}+{\sigma }_{j}\right)$$

26 $${\varepsilon }_{ij}=\sqrt{{\varepsilon }_{i}{\varepsilon }_{j}}\left(1-{k}_{ij}\right)$$

The $\rho \left( {{\text{\AA}}^{ - 3} } \right)$, is equal to:

27 $$\rho =\frac{6}{\pi }\eta {\left({\sum }_{i=1}^{N}{y}_{i}{m}_{i}{d}_{i}^{3}\right)}^{-1}$$

The compressibility factor is defined as:

28 $$Z=1+\eta {\left(\frac{\partial {\widetilde{a}}^{res}}{\partial \eta }\right)}_{T,{x}_{i}}=1+{Z}^{hc}+{Z}^{disp}$$

The pressure can be calculated as:

29 $$P=ZkT\rho {\left({10}^{10}\frac{A}{m}\right)}^{3}$$

The fugacity coefficient is defined by Eq. (30):

30 $$ln{\varnothing }_{k}=\frac{{\mu }_{k}^{res}\left(T, v\right)}{kT}-ln Z$$

The chemical potential can be calculated via Eq. (31):

31 $$\frac{{\mu }_{k}^{res}\left(T, v\right)}{kT}={\widetilde{a}}^{res}+\left(Z-1\right)+{\left(\frac{\partial {\widetilde{a}}^{res}}{\partial {x}_{k}}\right)}_{T,v,{x}_{j}\ne k}-\sum \left[{y}_{i}{\left(\frac{\partial {\widetilde{a}}^{res}}{\partial x}\right)}_{T,v,{x}_{i}\ne j}\right]$$

The amount of the segment number, energy, and diameter of CO_2_ and verapamil are obtained in Table 4.

**Table 4:** The PC-SAFT EoS parameters.

Material	*m*	$\sigma \left( {\text{\AA}} \right)$	$\frac{\varepsilon }{k}(K)$
CO_2_	2.07^a^	2.78^a^	169.21^a^
Verapamil	7.07	4.15	279.41

^a^Data for CO_2_ is obtained from^51^.

### Expanded liquid theory

Since the supercritical fluid density is close to that of a liquid, the supercritical fluid phase can be assumed to be an expanded liquid^34^. The pure solid and the supercritical phase have equilibrium^35^:

32 $${f}_{2}^{s}={f}_{2}^{SCF}={f}_{2}^{L}$$

where ${f}_{2}^{ScF}$ and ${f }_{2 }^{S}$ is the fugacity of verapamil in the supercritical phase and fugacity of the verapamil in the solid phase. The ${f}_{2}^{L}$ is defined as follows:

33 $${f}_{2}^{L}={\gamma }_{2}{y}_{2}{ f}_{2}^{0L}$$

Rewriting Eq. (31) will give the following result:

34 $${f}_{2}^{os}={\gamma }_{2}{y}_{2}{f}_{2}^{0L}$$

Here ${\gamma }_{2}$ and ${f}_{2}^{0L}$ are the activity coefficient and fugacity of the verapamil in the expanded liquid phase. The ratio ${f }_{2}^{0S}$ to ${f}_{2}^{0L}$ is calculated by Eq. (35) ^36^:

35 $$ln\frac{{f }_{2}^{\,0S}}{{f }_{2}^{0L}}=\frac{-\Delta {H }_{2 }^{\,f}}{R} \left(\frac{1}{T}-\frac{1}{{T}_{m}}\right)-\frac{\Delta Cp}{RT}\left(\frac{T- {T}_{m}}{T}\right)+\frac{\Delta Cp}{R} ln\left(\frac{T}{{T}_{m}}\right)$$

It can be written with Eqs. (33) and (34):

36 $${y}_{2}=\frac{1}{{\gamma }_{2}}\mathrm{exp}\left(\frac{-\Delta {H }_{2 }^{\,f}}{R} \left(\frac{1}{T}- \frac{1}{{T}_{m}}\right)\right)$$

Due to the very small value of verapamil in the supercritical fluid, the mole fraction is computed via Eq. (37):

37 $${y}_{2}=\frac{1}{{\gamma }_{2}^{\infty }}exp \left[\frac{-\Delta {H }_{2 }^{\,f}}{R} \left(\frac{1}{T}- \frac{1}{{T}_{m}}\right)\right]$$

#### Modified Wilson model

The Excess Gibbs energy ${G}^{E}$ can be calculated by Eq. (38) ^28^:

38 $$\frac{{G}^{E}}{RT}=-{y}_{1}ln\left({y}_{1}+{y}_{2}{\Lambda }_{12}\right)-{y}_{2}ln\left({y}_{1}{\Lambda }_{21}+{y}_{2}\right)$$

39 $${\Lambda }_{12}=\frac{{v}_{2}}{{v}_{1}}exp\left(-\frac{{\lambda }_{12}-{\lambda }_{11}}{RT}\right)$$

40 $${\Lambda }_{21}=\frac{{v}_{1}}{{v}_{2}}exp\left(-\frac{{\lambda }_{21}-{\lambda }_{22}}{RT}\right)$$

where ${v}_{1}$ and ${v}_{2}$ are the molar volume of SC-CO_2_ and verapamil. The activity coefficient of dilute verapamil in supercritical carbon dioxide is defined as^28^:

41 $$ln{\gamma }_{2}^{\infty }=1-{\Lambda }_{12}-ln\,ln {\Lambda }_{21}$$

42 $${\Lambda }_{12}={v}_{2}{\rho }_{c}{\rho }_{r}exp\left(-\frac{{\lambda^{\prime}}_{12}}{{T}_{r}}\right)$$

43 $${\Lambda }_{21}=\frac{1}{{v}_{2}{\rho }_{c}{\rho }_{r}}exp\left(-\frac{{\lambda^{\prime}}_{21}}{{T}_{r}}\right)$$

The ${\lambda {\prime}}_{12}$ and ${\lambda {\prime}}_{21}$ are as follows:

44 $${\lambda^{\prime}}_{12}=\frac{{\lambda }_{12}}{R{T}_{c1}}$$

45 $${\lambda^{\prime}}_{21}=\frac{{\lambda }_{21}}{R{T}_{c1}}$$

The molar volume ${(\upsilon }_{2})$ is defined as Eq. (46) ^37^:

46 $${\upsilon }_{2}=\alpha \,{\rho }_{r}+\beta$$

These four parameters ($\alpha$, $\beta$, ${\lambda^{\prime}}_{12}$ and ${\lambda^{\prime}}_{21}$ ) are determined by the model.

### Regular solution model

The fugacity of each element in the two phases must be equal to be in equilibrium. The solubility of carbon dioxide in the solid phase is considered negligible. The activity of the verapamil in the liquid phase is equal to the fugacity of the pure solute in the liquid phase in solid–liquid equilibrium. Fugacity can be calculated by knowing the solid substance's enthalpy and melting point. With the use of a solution model and the Flory–Huggins based, the activity coefficient is calculated. So, the solubility of solute (verapamil) in SC-CO_2_ is calculated using the Eq. (47).

47 $$ln {y}_{2}=\frac{\Delta {H }_{2 }^{m}}{RT} \left(\frac{T}{{T}_{m}}- 1\right)- \frac{{v}_{2}}{RT} {\left({\delta }_{1}-{\delta }_{2}\right)}^{2}- ln\left(\frac{{v}_{2}}{{v}_{1}}\right)-1+\frac{{v}_{2}}{{v}_{1}}$$

where $\Delta {H }_{2 }^{m}$ is heat fusion and ${\delta }_{1}$ is the SC-CO_2_ solubility parameter. It is calculated by Eq. (48):

48 $${\delta }_{1}^{2}={\left[\frac{{\delta }_{dref}}{{\left(\frac{{v}_{ref}}{{v}_{1}}\right)}^{-1.25}}\right]}^{2}+{\left[\frac{{\delta }_{pref}}{{\left(\frac{{v}_{ref}}{{v}_{1}}\right)}^{-0.5}}\right]}^{2}+{\left[\frac{{\delta }_{href}}{exp\left\{-1.32\times {10}^{-3}\left({T}_{ref}-T\right)-ln{\left(\frac{{v}_{ref}}{{v}_{1}}\right)}^{0.5}\right\}}\right]}^{2}$$

The values of *T*_*ref*_*,* ${\delta }_{pref}$, ${\delta }_{href},$ ${v}_{ref}$, and ${\delta }_{dref}$ are 298.15 K, $5.2 {\mathrm{MPa}}^\frac{1}{2}$, $5.8 {\mathrm{MPa}}^\frac{1}{2}$, $39.13$ cm^3^/mol, and $15.6 {\mathrm{MPa}}^\frac{1}{2},$ respectively^38^.

The solubility parameter of the solid solute is defined as:

49 $${\delta }_{2}= A+B{\rho }_{r,1}$$

50 $${\delta }_{2}= A+B{\rho }_{r,1}^{c}$$

51 $${\delta }_{2}= A+B{\rho }_{r,1}+C{\rho }_{r,1}^{2}$$

${\rho }_{r,1}$ is the reduced density of the supercritical fluid.

## Results and discussion

### Experimental data

**Figure 2:**
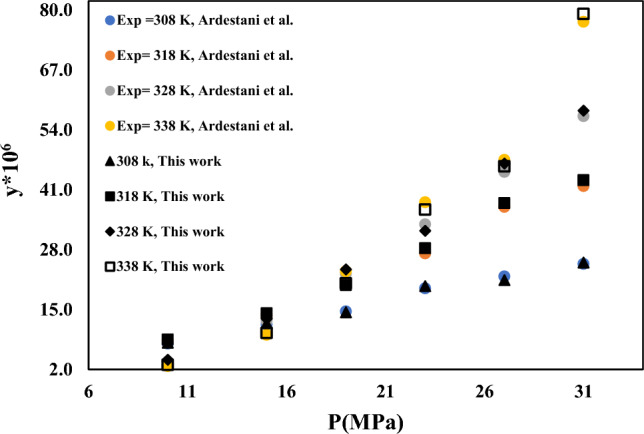
Experimental solubility data of Capecitabine in SC–CO_2_ and comparison with Ardestani et al. data^39^.

**Figure 3:**
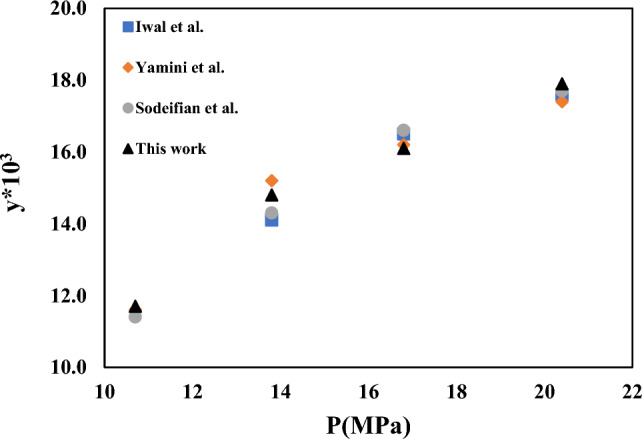
Experimental solubility data of naphthalene in SC-CO_2_ and comparison with Iwai et al. ^40^, Yamini et al. ^41^, and Sodeifian et al. ^42^ data.

**Figure 4:**
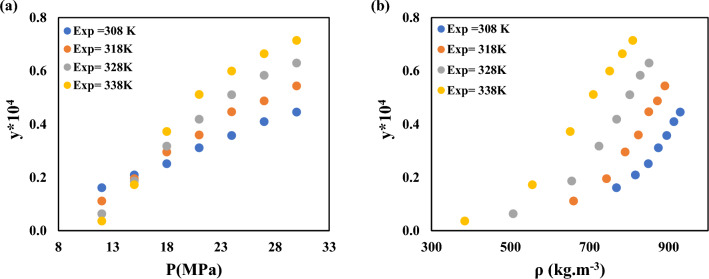
The effect of (**a**) pressure and (**b**) density of SC-CO_2_ on verapamil solubility at several temperatures.

In order to demonstrate the reliability of the solubility measurement equipment, the solubility of capecitabine and naphthalene at different temperatures and pressures are measured using the device used in this study and compared with the data reported for Ardestani et al.^39^, Iwai et al.^40^, Yamini et al.^41^, Sodeifian et al.^42^. Experimental data of capecitabine and naphthalene in SC-CO_2_ are compared in Figs. 2 and 3. Table 5 provides information on verapamil's solubility in SC-CO_2_. At temperatures between 308 and 338 K and pressures between 12 and 30 MPa, the SC-CO_2_ density, mole fraction, and verapamil solubility are all tested in triplicate. The density of SC-CO_2_ is determined by Span-Wanger EoS^43^. Based on the NIST recommendation, combined and extended uncertainties are presented in Table 5. Figure 4 depicts how SC-CO_2_ pressure and density affect the solubility of verapamil. Verapamil's solubility in SC-CO_2_, as seen in Table 5 and Fig. 4, increased as pressure increased because verapamil's solvent density and vapor pressure rose as temperature and pressure rise. The crossover zone for verapamil's solubility is between 12 and 15 MPa. In this zone, the solubility of verapamil in SC-CO_2_ is reduced with temperature increase in this range. On the contrary, the solubility of verapamil increased when the temperature is over this range. The struggle between the impacts of verapamil vapor pressure and CO_2_ density on temperature led to the crossover. The sublimation pressure and enthalpy, critical characteristics, and solute molar volume are only a few of the variables that might affect the cross-over point^44,45^. The solubility of verapamil in SC-CO_2_ expressed as mole fraction is in the border of 3.6 × 10^–6^ to 7.14 × 10^–5^, which are collected at the temperature of 338 K and pressures of 12 and 30 MPa, respectively.

**Table 5:** The verapamil solubility test data in SC-CO_2_. The *y*_2_ and *S* are mole fractions of verapamil and solubility of verapamil.^a^

Temperature^b^ (K)	Pressure^b^ (MPa)	Density^c^ (kg/m^3^)	Binary			
			*y*_2_ × 10^4^ (Mole fraction )	Standard deviation of the mean, SD(ȳ) × (10^5^)	Expanded uncertainty × 10^6^	*S* × 10 (Solubility (g/l))
308	12	768	0.161	0.026	0.521	1.279
	15	816	0.209	0.066	1.321	1.765
	18	849	0.251	0.057	1.142	2.204
	21	874	0.311	0.082	1.642	2.812
	24	896	0.357	0.112	2.242	3.305
	27	914	0.409	0.145	2.902	3.863
	30	930	0.445	0.169	3.382	4.275
318	12	660	0.111	0.026	0.521	0.758
	15	743	0.195	0.054	1.081	1.500
	18	790	0.295	0.089	1.781	2.411
	21	824	0.359	0.123	2.461	3.058
	24	850	0.446	0.175	3.502	3.920
	27	872	0.487	0.166	3.322	4.390
	30	891	0.543	0.206	4.122	4.999
328	12	507	0.063	0.015	0.300	0.332
	15	655	0.186	0.037	0.741	1.261
	18	724	0.317	0.072	1.442	2.375
	21	769	0.418	0.111	2.222	3.323
	24	802	0.510	0.155	3.102	4.230
	27	829	0.583	0.133	2.663	4.992
	30	851	0.629	0.173	3.463	5.530
338	12	384	0.036	0.011	0.220	0.144
	15	555	0.172	0.059	1.181	0.991
	18	651	0.372	0.141	2.821	2.507
	21	710	0.511	0.136	2.723	3.750
	24	751	0.599	0.182	3.643	4.651
	27	783	0.664	0.227	4.543	5.375
	30	810	0.714	0.271	5.423	5.973

^a^The experimental standard deviation and the experimental standard deviation of the mean (SD) are computed by $S\left({y}_{k}\right)=\sqrt{\frac{{\sum }_{j=1}^{n}{\left({y}_{i}-y\right)}^{2}}{n-1}}$ and $SD\left(\underline{y}\right)=\frac{S\left({y}_{k}\right)}{\sqrt{n}}$ respectively. The relative combined standard uncertainty is earned by ${U}_{combined}/y=\sqrt{{\sum }_{i=1}^{N}{\left({P}_{i}U\left({x}_{i}\right)/{x}_{i}\right)}^{2}}$. The expanded uncertainty *U* is $k\times {U}_{combined}$. ^b^Standard uncertainty u are (T) = 0.1 K; u(p) = 0.1 MPa. The relative standard uncertainties are computed below 0.05 for solubilities and mole fractions.

### Solubility data with semi-empirical models

**Figure 5:**
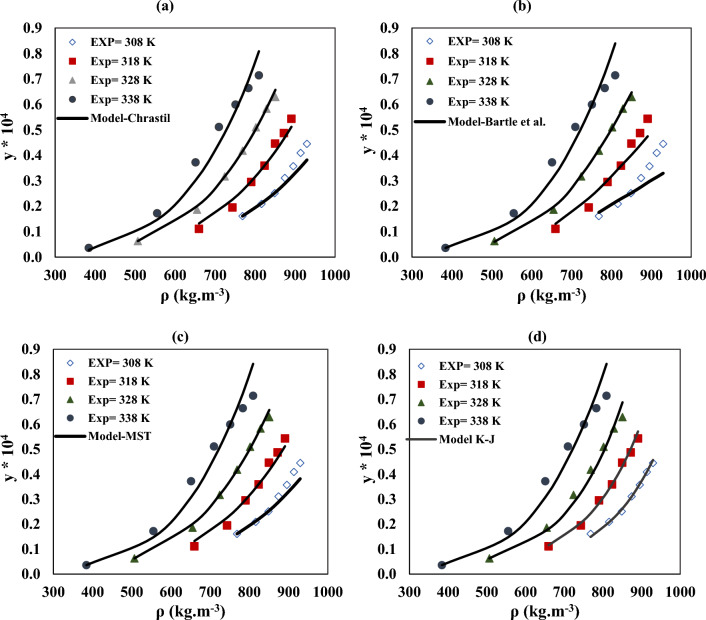
The experimental and model values of verapamil solubility according to the (**a**) Chrastil, (**b**) Bartle et al., (**c**) MST, and (**d**) K–J., models at different temperatures.

To correlate the experimental data of verapamil solubility in SC-CO_2_, semi-empirical density-based models (Chrastil, Bartle et al., MST, and K–J) are proposed in this study. The adjustable parameters of the semi-empirical model (*a*_0_*, a*_1_*, a*_2_), *AARD%*, and *R*_*adj*_ are reported in Table 6. The empirical and theoretical solubility data of verapamil obtained by the semi-empirical models are shown in Fig. 5. As indicated in *AARD%* in Table 6, the best models are K–J, MST, Chrastil et al., and Bartle, respectively. The review of other studies regarding the solubility of pharmaceutical substances shows that model K–J is reported to be the best in many cases such as salsalate^46^, Favipiravir^18^, and Lacosamide^19^. MST models also presented acceptable capability to describe the solubility of verapamil in SC-CO_2_. Total, vaporization solvation enthalpy is calculated with the tuning parameter of the Chrastil (${a}_{1}$) and Bartle et al. (${a}_{2}$) model. The values of these enthalpy are listed in Table 7.

**Table 6:** The semi-empirical model's results of the verapamil– CO_2_ system.^a^

Model	${a}_{0}$	${a}_{1}$	${a}_{2}$	*AARD%*	*R*_*adj*_
Chrastil	5.5	− 4766.3	− 23.2	8.20	0.9830
Bartle et al	16.63	0.00954	− 7220.83	9.93	0.9600
MST	− 9875.4	3.2	17.8	7.90	0.9721
K–J	− 0.8	0.3	− 4831.7	7.48	0.9742

^a^a_0_ − a_2_, adjustable parameters of models.

**Table 7:** Enthalpy for verapamil.

Compound	ΔH_total_ (kJ/mol)^a^	ΔH_vap._ (kJ/mol)^b^	ΔH_sol._ (kJ/mol)^c^
Verapamil	39.62	60.03	− 20.41

^a^Chrastil's procedure.

^b^Bartle et al*.*, procedure

^c^difference between the Δ*H*_*vap*_ and Δ*H*_*total*_*.*

### Solubility correlation with the equation of state model

**Figure 6:**
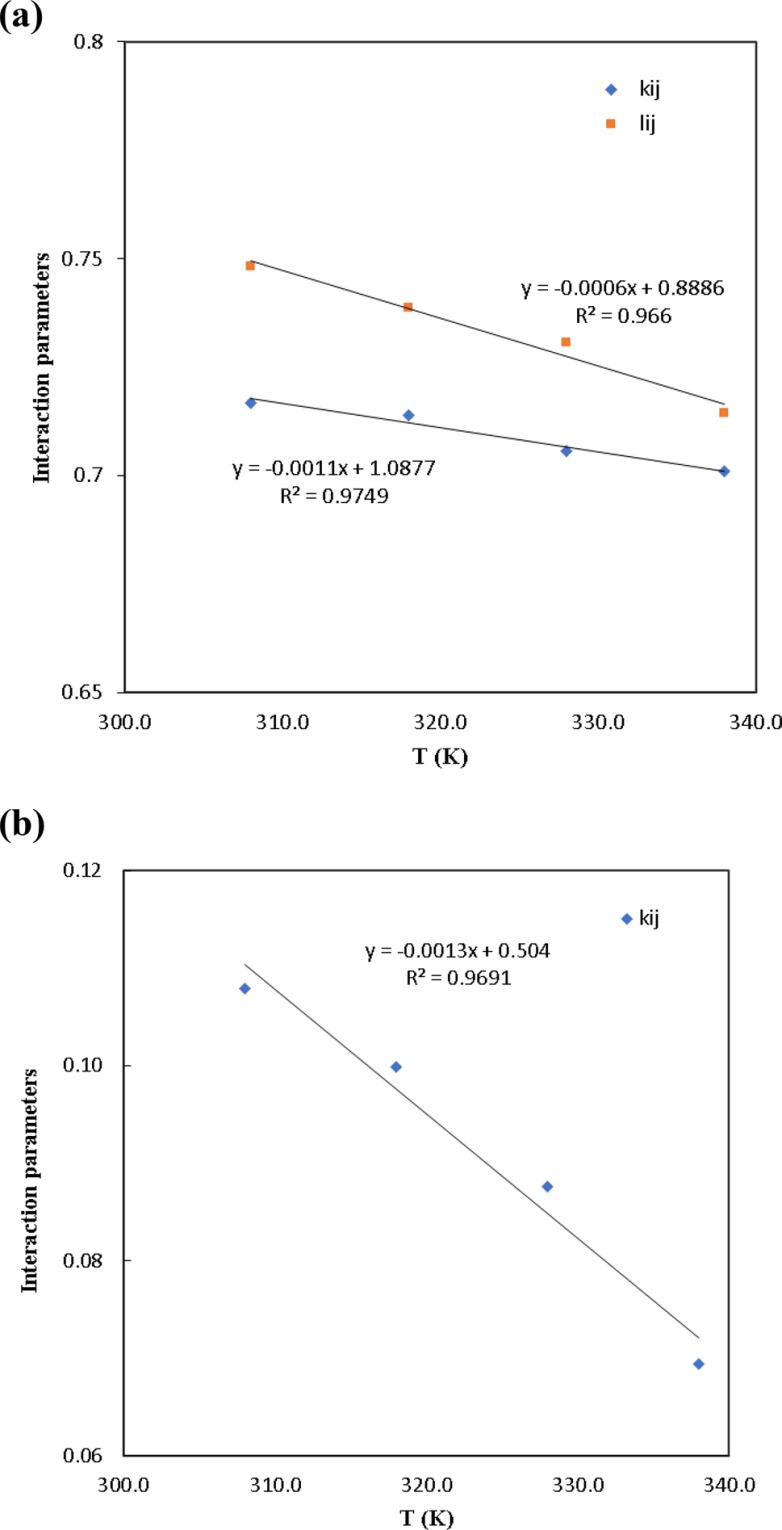
The effect of temperature on the interaction parameters for the verapamil-CO_2_ system; (**a**) SRK, (**b**) PC-SAFT.

A simulated annealing method is used to improve the interaction parameters of the SRK EoS and PC-SAFT. The adjustable parameters of PR-EoS (${k}_{ij}$, ${l}_{ij}$) and PC-SAT (${k}_{ij}$) are dependent temperature and represented in Table 8. Figure 6 depicts how temperature affects the interaction parameters for the binary system verapamil-SC-CO_2_ by SRK and PC-SAFT EoS models. The relation between interaction parameters and temperature is defined as follows:

**Table 8:** Solidarity outcomes for solubility of verapamil in SC-CO_2_, by SRK and PC-SAFT.

Model	Parameter	T = 308 K	T = 318 K	T = 328 K	T = 338 K	Overall
SRK	${k}_{12}$	0.7167	0.7140	0.7056	0.7010	
	${l}_{12}$	0.7483	0.7387	0.7306	0.7144	
	$AARD$	5.43	20.05	30.37	26.89	20.68
	${R}_{adj}$	0.9581	0.9391	0.9281	0.9192	0.9361
PC-SAFT	${k}_{12}$	0.1075	0.0995	0.0871	0.0687	
	$AARD$	6.34	8.87	6.78	7.98	7.45
	${R}_{adj}$	0.9712	0.9695	0.9764	0.9521	0.9588

52 $${l}_{ij}=AT+B$$

53 $${k}_{ij}=CT+D$$

**Figure 7:**
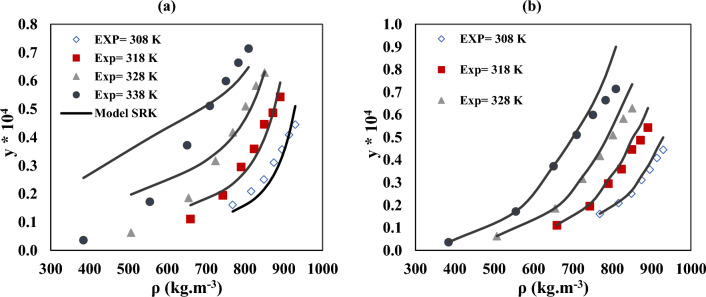
The experimental and model values solubility of verapamil according to (**a**) SRK and (**b**) PC-SAT at several temperatures.

Figure 6 illustrates how this relationship's slope and intercept are identified using linear regression analysis. Figure 7 displays the empirical and predicted solubility data by the SRK and PC-SAFT EoS model at 308, 318, 328, and 338 K. SRK and PC-SAFT both had total *AARD%* of 20.68 and 7.45, respectively. As indicated by the *AARD%* in Table 8, while determining the solubility of verapamil in SC-CO_2_, PC-SAFT showed a higher verification of the SRK EoS.

### Expanded liquid theory-modified Wilson model

**Figure 8:**
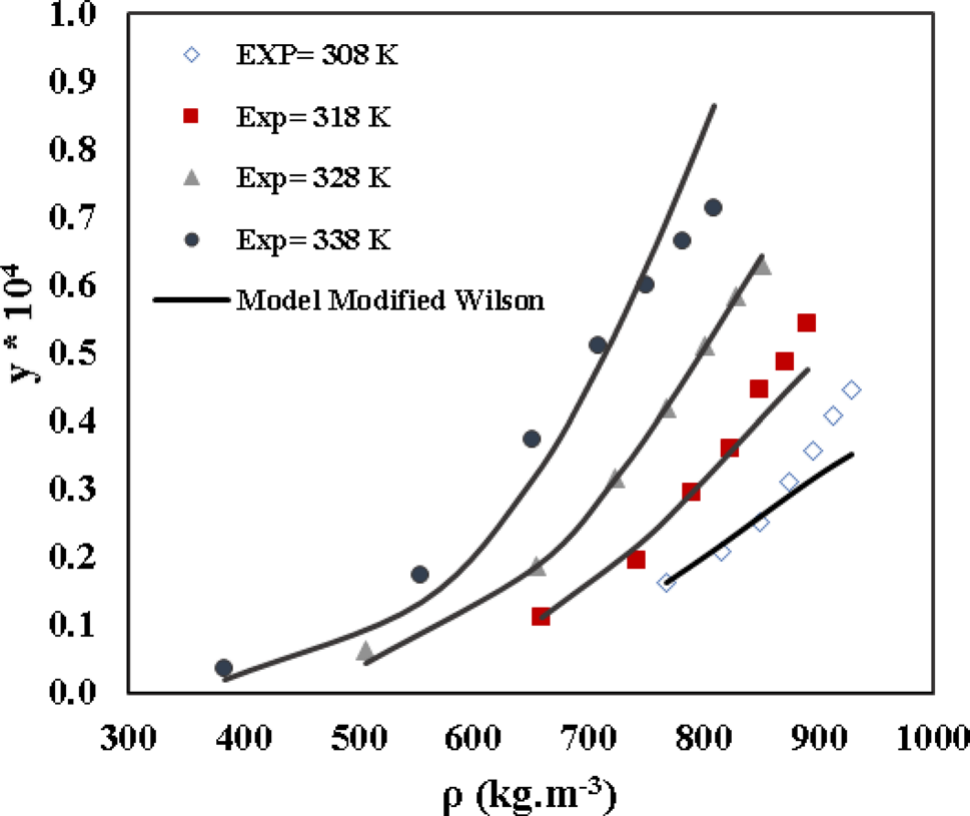
The experimental data and model solubility of verapamil in SC-CO_2_ according to modified Wilson.

The model parameters for the solubility of verapamil in SC-CO_2_ are optimized using the modified Wilson model. Table 9 lists the parameters of the modified Wilson model ($\alpha$, $\beta$, ${\lambda {\prime}}_{12}$, and ${\lambda {\prime}}_{21}$). The modified Wilson model's ability to forecast the solubility of verapamil in SC-CO_2_ is demonstrated by the value of *AARD%*, which is 9.89. The value of ${\lambda {\prime}}_{12}$ (− 2.0196) is smaller than ${\lambda {\prime}}_{21}$ (17.0059). So, according to Eqs. (39) and (40) the value of ${\Lambda }_{21}$ is calculated to be smaller than ${\Lambda }_{12}$. The testing data and calculated solubility of verapamil in SC-CO_2_ with a modified Wilson model are seen in Fig. 8.

**Table 9:** Modified Wilson parameters for solubility of verapamil in SC-CO_2_.

$\alpha$	$\beta$	${\lambda {\prime}}_{12}$	${\lambda {\prime}}_{21}$	*AARD%*	*R*_*adj*_
− 0.00003806	0.00001696	− 2.0196	17.0059	9.89	0.9621

### Regular solution model

**Figure 9:**
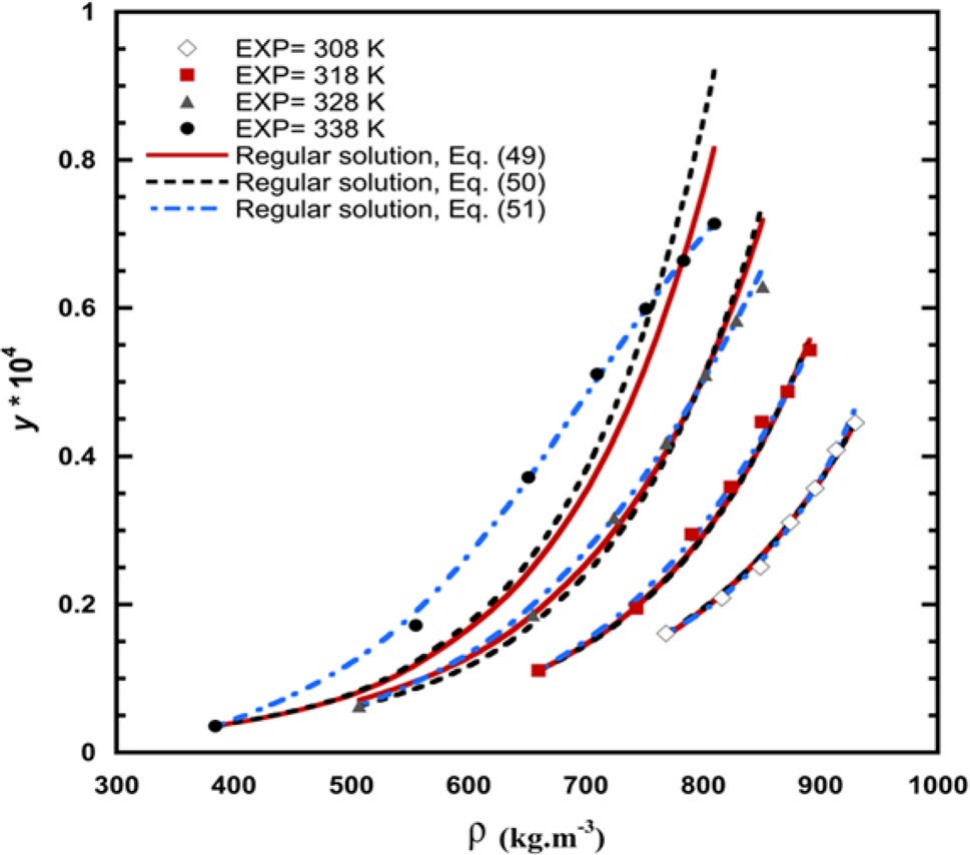
The experimental data and model solubility of verapamil in SC-CO_2_ according to the regular solution.

Table 10 contains the results of the correlation for the solubility of verapamil in SC-CO_2_. The *A, B,* and *C* are adjustable parameters of Eqs. (49)–(51). The values of tuning parameters (A, B, and C) are calculated with higher veracity at low temperatures (Table 10). The *AARD%* and *R*_*adj*_ for Eqs. (49)–(51) are determined and obtained in Table 10. As indicated by *AARD%*, Eq. (51) showed higher veracity in calculating the solubility of verapamil in SC-CO_2_. The experimental data and predicted solubility of verapamil in SC-CO_2_ based on a regular solution model are shown in Fig. 9.

**Table 10:** Regular solution models outcomes for solubility of verapamil in Sc-CO_2_.

Regular solution model	*T*/(K)	Correlation parameters			*AARD/%*	*R*_*adj*_
		*A*	*B*	*C*		
Equation (49)	308	− 7498.9	− 357		1.62	0.9958
	318	− 7970.4	− 242.41		3.03	0.9891
	328	− 8165.5	− 251.45		5.90	0.9789
	338	− 8660.1	− 104.8		17.30	0.9013
	Overall	6.96	0.9664			
Equation (50)	308	12,471	− 20,216	0.033003	1.51	0.9962
	318	− 210.49	− 7969	0.048068	3.21	0.9874
	328	− 911.32	− 7550.5	0.032842	6.77	0.9700
	338	− 14,566	5806.3	− 0.01174	17.90	0.8907
	Overall	7.34	0.9611			
Equation (51)	308	− 5383.2	− 2720.1	657.13	1.26	0.9952
	318	− 8979.4	1013.8	− 383.49	1.99	0.9953
	328	− 9188.1	1182.3	− 489.66	1.73	0.9971
	338	− 10,025	2371.3	− 991.1	1.77	0.9990
	Overall	1.68	0.9966

### Comparison of all models used

**Figure 10:**
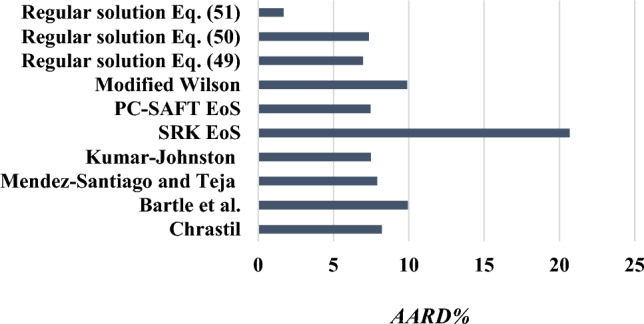
Comparison of the *AARD%* calculated for all models (semi-empirical density-based, equations of state, expanded liquid, and regular solution).

**Figure 11:**
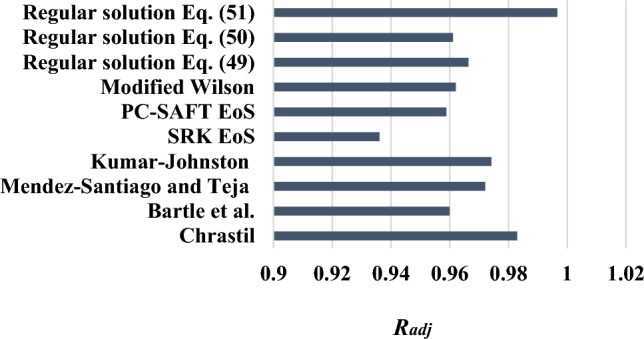
Comparison of the *R*_*adj*_ calculated for all models (semi-empirical density-based, equations of state, expanded liquid, and regular solution).

The experimental solubility of verapamil is measured at different temperature and pressure values. The experimental solubility data are correlated with four semi-empirical density-based models (Chrastil, Bartle, Kumar-Johnston (K–J), and Mendez-Santiago and Teja (MST)), two equations of state (SRK and PC-SAFT EoS), expanded liquid models (modified Wilson's models), and regular solution model. Figure 10 shows the *AARD%* of all the models used in this research. According to Fig. 10, regular solution with different Eqs. (49), (50), and (51) are the best methods with minimum *AARD*%. The worst is SRK EoS. Otherwise, the PC-SAFT EoS method is more accurate in comparison to another method. Also, models are compared by *R*_*adj*_ in Fig. 11. The best model has the highest *R*_*adj*_. The regular solution is the best method, based on *AARD%* and *R*_*adj*_. The SRK EoS is the worst method, based on *AARD%* and *R*_*adj*_.

## Conclusion

Experimental measurements are developed to determine the solubility of verapamil in SC-CO_2_ at various pressure and temperature values. The lowest determined mole fraction of verapamil in SC-CO_2_ is 3.6 × 10^–6^ at 338 K and 12 MPa. Verapamil's experimental solubility data in SC-CO_2_ are correlated using the Chrastil, Bartle et al., MST, and K–J methods. The K–J model with *AARD%* 7.48 appears better contracted with solubility data of verapamil when compared to the other semi-empirical models. Comparing the SRK and PC-SAFT EoS revealed that the PC-SAFT is more accurate in determining the solubility of verapamil in SC-CO_2_ than the SRK EoS. The parameters of SRK-EoS (${k}_{ij}$, ${l}_{ij}$) and PC-SAT (${k}_{ij}$) are determined. According to the results, regular solution model Eq. (51) (*AARD%* = 1.68), and PC-SAFT EoS (*AARD%* = 7.45) presented a better contracted with solubility data of verapamil when compared to the semi-empirical model K–J (*AARD%* = 7.48), MST (*AARD%* = 7.90), Chrastil (*AARD%* = 8.20), modified Wilson model (*AARD%* = 9.89), Bartle et al*.* (*AARD%* = 9.93), and SRK EoS (*AARD%* = 20.68). The poor solubility of verapamil in SC-CO_2_ is in the range of 3.6 × 10^–6^ to 7.14 × 10^–5^ that confirms supercritical anti-solvent methods can be an appropriate choice to produce nanoparticles of this drug.

## Data Availability

The datasets used and/or analyzed during the current study are available from the corresponding author on reasonable request.

## References

[1] Eteraf-Oskouei T, Mikaily Mirak S, Najafi M (2017). Anti-inflammatory and anti-angiogenesis effects of verapamil on rat air pouch inflammation model. Adv. Pharm. Bull..

[2] Agrawal YO (2022). Verapamil hydrochloride loaded solid lipid nanoparticles: Preparation, optimization, characterisation, and assessment of cardioprotective effect in experimental model of myocardial infarcted rats. Biomed. Pharmacother..

[3] O'Neil MJ (2001). The Merck Index: An Encyclopedia of Chemicals, Drugs, and BIOLOGICALS.

[4] Esfandiari N (2015). Production of micro and nano particles of pharmaceutical by supercritical carbon dioxide. J. Supercrit. Fluids.

[5] Najafi M, Esfandiari N, Honarvar B, Arab-Aboosadi Z (2021). Experimental investigation on finasteride microparticles formation via gas antisolvent process. Korean Chem. Eng. Res..

[6] Sodeifian G, Saadati Ardestani N, Sajadian SA, Soltani-Panah H (2019). Experimental measurements and thermodynamic modeling of Coumarin-7 solid solubility in supercritical carbon dioxide: Production of nanoparticles via RESS method. Fluid Phase Equil..

[7] Chen B-Q (2022). Preparation of astragaloside IV (AS-IV) nanoparticles via SAS process for anticancer efficacy: Optimization based on Box-Behnken design. J. Supercrit. Fluids.

[8] Santos AE, Dal Magro C, de Britto LS, Aguiar GPS, de Oliveira JV, Lanza M (2022). Micronization of luteolin using supercritical carbon dioxide: Characterization of particles and biological activity in vitro. J. Supercrit. Fluids.

[9] Najafi M, Esfandiari N, Honarvar B, Arab-Aboosadi Z (2020). Thermodynamic modeling of the gas-antisolvent (GAS) process for precipitation of finasteride. J. Chem. Petrol. Eng..

[10] Najafi M, Esfandiari N, Honarvar B, Arab-Aboosadi Z (2021). Production of rosuvastatin calcium nanoparticles using gas antisolvent technique: Experimental and optimization. Period. Polytech. Chem. Eng..

[11] Esfandiari N, Sajadian SA (2022). CO2 utilization as gas antisolvent for the pharmaceutical micro and nanoparticle production: A review. Arab. J. Chem..

[12] Kaga K (2018). Nanoparticle formation of PVP/astaxanthin inclusion complex by solution-enhanced dispersion by supercritical fluids (SEDS): Effect of PVP and astaxanthin Z-isomer content. J. Supercrit. Fluids.

[13] Islam T, Sarker MZI, Uddin ABMH, Smith RL (2022). Acetaminophen synthesis and encapsulation using safe mixed-solvents and solution enhanced dispersion by supercritical CO2. J. Supercrit. Fluids.

[14] Kumar R (2022). Numerical simulation to estimate the droplet size in aerosol solvent extraction system. Mater. Today: Proc..

[15] Yan T, Zhang Y, Ji M, Wang Z, Yan T (2019). Preparation of irbesartan composite microparticles by supercritical aerosol solvent extraction system for dissolution enhancement. J. Supercrit. Fluids.

[16] Tokunaga S (2021). Microencapsulation of drug with enteric polymer Eudragit L100 for controlled release using the particles from gas saturated solutions (PGSS) process. J. Supercrit. Fluids.

[17] Esfandiari N, Sajadian SA (2022). Experimental and modeling investigation of Glibenclamide solubility in supercritical carbon dioxide. Fluid Phase Equilib..

[18] Sajadian SA, Ardestani NS, Esfandiari N, Askarizadeh M, Jouyban A (2022). Solubility of favipiravir (as an anti-COVID-19) in supercritical carbon dioxide: An experimental analysis and thermodynamic modeling. J. Supercrit. Fluids.

[19] Esfandiari N, Ali Sajadian S (2022). Solubility of Lacosamide in supercritical carbon dioxide: An experimental analysis and thermodynamic modeling. J. Mol. Liq..

[20] Amani M, Ardestani NS, Jouyban A, Sajadian SA (2022). Solubility measurement of the fludrocortisone acetate in supercritical carbon dioxide: Experimental and modeling assessments. J. Supercrit. Fluids.

[21] Sadeghi M, Cascella F, Tenberg V, Seidel-Morgenstern A, Lorenz H (2021). Solubility analysis of pharmaceuticals guaifenesin, ketoprofen, and artemisinin in different solvents. J. Mol. Liq..

[22] Jouyban A, Rehman M, Shekunov BY, Chan H-K, Clark BJ, York P (2002). Solubility prediction in supercritical CO2 using minimum number of experiments. J. Pharm. Sci..

[23] Garlapati C, Madras G (2010). New empirical expressions to correlate solubilities of solids in supercritical carbon dioxide. Thermochim. Acta.

[24] Mackay, D., Boethling, R. S. *Handbook of Property Estimation Methods for Chemicals: Environmental Health Sciences*. (CRC Press, 2000).

[25] Immirzi A, Perini B (1977). Prediction of density in organic crystals. Acta Crystallogr. Sect. A.

[26] Marrero J, Gani R (2001). Group-contribution based estimation of pure component properties. Fluid Phase Equil..

[27] Poling, B. E., Prausnitz, J. M., OConnell, J.P. *The properties of gases and liquids*. 5th edn (McGraw-Hill, New York, 2001).

[28] Soave G (1972). Equilibrium constants from a modified Redlich-Kwong equation of state. Chem. Eng. Sci..

[29] Gross J, Sadowski G (2000). Application of perturbation theory to a hard-chain reference fluid: An equation of state for square-well chains. Fluid Phase Equil..

[30] Azim MM, Ushiki I, Miyajima A, Takishima S (2022). Modeling the solubility of non-steroidal anti-inflammatory drugs (ibuprofen and ketoprofen) in supercritical CO2 using PC-SAFT. J. Supercrit. Fluids.

[31] Azim MM, Ushiki I, Miyajima A, Takishima S (2022). Estimating the solubility of salsalate in supercritical CO2 via PC-SAFT modeling using its experimental solubility data in organic solvents. J. Supercrit. Fluids.

[32] Hadj A, Si-Moussa C, Hanini S, Laidi M (2013). Application of PC-SAFT and cubic equations of state for the correlation of solubility of some pharmaceutical and statin drugs in SC-CO2. Chem. Ind. Chem. Eng. Q..

[33] Abdallah-El-Hadj A, Si-Moussa C, Hanini S, Laidi M (2013). Application of PC-SAFT and cubic equations of state for the correlation of solubility of some pharmaceutical and statin drugs in SC-CO2. Chem. Ind. Chem. Eng. Quart..

[34] Higashi H, Iwai Y, Arai Y (2001). Solubilities and diffusion coefficients of high boiling compounds in supercritical carbon dioxide. Chem. Eng. Sci..

[35] Nasri L (2018). Modified Wilson's model for correlating solubilities in supercritical fluids of some polycyclic aromatic solutes. Polycycl. Aromat. Compd..

[36] Prausnitz, J. M., Lichtenthaler, R.N., de Azevedo, E.G. *Molecular thermodynamics of fluid-phase equilibria*. (Pearson Education, 1998).

[37] Tamura K, Alwi RS, Tanaka T, Shimizu K (2017). Solubility of 1-aminoanthraquinone and 1-nitroanthraquinone in supercritical carbon dioxide. J. Chem. Thermodyn..

[38] Williams LL, Rubin JB, Edwards HW (2004). Calculation of Hansen solubility parameter values for a range of pressure and temperature conditions, including the supercritical fluid region. Ind. Eng. Chem. Res..

[39] Ardestani NS, Majd NY, Amani M (2020). experimental measurement and thermodynamic modeling of capecitabine (an anticancer drug) solubility in supercritical carbon dioxide in a ternary system: Effect of different cosolvents. J. Chem. Eng. Data.

[40] Iwai Y, Fukuda T, Koga Y, Arai Y (1991). Solubilities of myristic acid, palmitic acid, and cetyl alcohol in supercritical carbon dioxide at 35degree.C. J. Chem. Eng. Data.

[41] Yamini Y, Fat'hi MR, Alizadeh N, Shamsipur M (1998). Solubility of dihydroxybenzene isomers in supercritical carbon dioxide. Fluid Phase Equilibria.

[42] Sodeifian G, Sajadian SA, Ardestani NS (2017). Determination of solubility of Aprepitant (an antiemetic drug for chemotherapy) in supercritical carbon dioxide: Empirical and thermodynamic models. J. Supercrit. Fluids.

[43] Span R, Wagner W (1996). A new equation of state for carbon dioxide covering the fluid region from the triple-point temperature to 1100 K at pressures up to 800 MPa. J. Phys. Chem. Ref. Data.

[44] Kalikin NN, Oparin RD, Kolesnikov AL, Budkov YA, Kiselev MG (2021). A crossover of the solid substances solubility in supercritical fluids: What is it in fact?. J. Mole. Liquids.

[45] Vieira de Melo SAB, Costa GMN, Viana ACC, Pessoa FLP (2009). Solid pure component property effects on modeling upper crossover pressure for supercritical fluid process synthesis: A case study for the separation of Annatto pigments using SC-CO2. J. Supercrit. Fluids.

[46] Zabihi S (2021). Measuring salsalate solubility in supercritical carbon dioxide: Experimental and thermodynamic modelling. J. Chem. Thermodyn..

[47] Chrastil J (1982). Solubility of solids and liquids in supercritical gases. J. Phys. Chem..

[48] Bartle KD, Clifford AA, Jafar SA, Shilstone GF (1991). Solubilities of solids and liquids of low volatility in supercritical carbon dioxide. J. Phys. Chem. Ref. Data.

[49] Méndez-Santiago J, Teja AS (1999). The solubility of solids in supercritical fluids. Fluid Phase Equilib..

[50] Kumar SK, Johnston KP (1988). Modelling the solubility of solids in supercritical fluids with density as the independent variable. J. Supercrit. Fluids.

[51] Gross J, Sadowski G (2001). Perturbed-chain SAFT: An equation of state based on a perturbation theory for chain molecules. Ind. Eng. Chem. Res..

